# An Aneurysm of the Internal Iliac Vein and Its Confluent Branches Due to Congenital Arteriovenous Communication: A Case Report

**DOI:** 10.7759/cureus.74726

**Published:** 2024-11-29

**Authors:** Nektarios Galanis, Antreas Dousis, Nikolaos Taprantzis, Dimosthenis Chrysikos, Ameer Shehade, Theodore Troupis

**Affiliations:** 1 Department of Anatomy, National and Kapodistrian University of Athens School of Medicine, Athens, GRC

**Keywords:** angiography imaging, arteriovenous communication, endovascular treatment (evt), internal iliac vein aneurysm, vascular disease

## Abstract

Iliac vein aneurysms are a relatively rare clinical medical case requiring careful management. In general, vessel aneurysms are always an intriguing entity to approach due to the various options for diagnosis and treatment, which are heavily dependent on the unique characteristics of the condition. Such features include etiology, location, and coexistence with other abnormalities. In this case report, we describe the case of a patient who presented with an aneurysm in his internal iliac vein, as well as its tributaries, as a result of congenital arteriovenous communication.

## Introduction

The internal iliac vein is a short vessel found in the pelvis that arises from several smaller veins just above the greater sciatic foramen [1]. From an embryological perspective, following the fifth week of development, the posterior branches of the cardinal veins are eliminated, with only the most caudal part remaining. This gives rise to the common iliac vein and the most caudal segment of the inferior vena cava, called the sacral segment [2]. Its main function is to drain the blood from the pelvic organs and pelvic wall, perineum and external genitalia, gluteal region, and the medial region of the thigh. It has a short superior course over the sacroiliac joint, after which it converges with the external iliac vein, forming the common iliac vein [3]. The internal iliac vein is formed by the confluence of several extrapelvic and pelvic veins. Its extrapelvic tributaries are the superior gluteal, inferior gluteal, internal pudendal, and obturator veins, and its pelvic tributaries are the middle rectal, vesical, uterine, and vaginal veins. Although there are many individual variations concerning the course of the venous plexuses and small veins that converge to form the internal iliac vein, their diameter is usually smaller than the internal iliac vein itself. The internal iliac vein has at its origin a diameter between 4.7 and 9.9 mm and at the end a diameter between 5.9 and 10.2 mm [4].

Statistically speaking, arteriovenous malformations are generally characterized as an uncommon occurrence. Studies have reported various data, from 0.89 to 1.34 cases per 100,000 persons, while other studies have reported 18 cases per 100,000 people [5]. On the other hand, there are not many studies that report statistics regarding the prevalence of arteriovenous fistulas in general. However, according to the National Institute of Diabetes and Digestive and Kidney Disease, up to 2013, more than 468,000 patients were on hemodialysis, with 20% of those receiving it through the creation of an arteriovenous fistula. Finally, in the HEMO study published in 2000, the prevalence of arteriovenous fistulas differed among dialysis centers between 4% and 77% [6].

We present a rare case of an enormous meandering aneurysm of the right internal iliac vein and its tributaries most likely due to congenital arteriovenous communication. Informed consent was obtained from the patient to present his case in an anonymized form to experts in the field.

## Case presentation

A 40-year-old male presented two years ago to the urology outpatient clinic with dull and intermittent pain in the right side of the pelvis accompanied by frequent urination and nocturia. There was no history of trauma or recent infections. The patient also suffered from osteoporosis for which he was being treated with denosumab. The urologically oriented clinical examination showed slight tenderness in the right lower abdomen and the symphysis pubis without any other pathological findings. The inflammatory parameters were negative. The ultrasound revealed a large venous mass to the right of the bladder which was slightly pushed medially. Based on these findings, a prompt CT angiography was planned.

**Figure 1:**
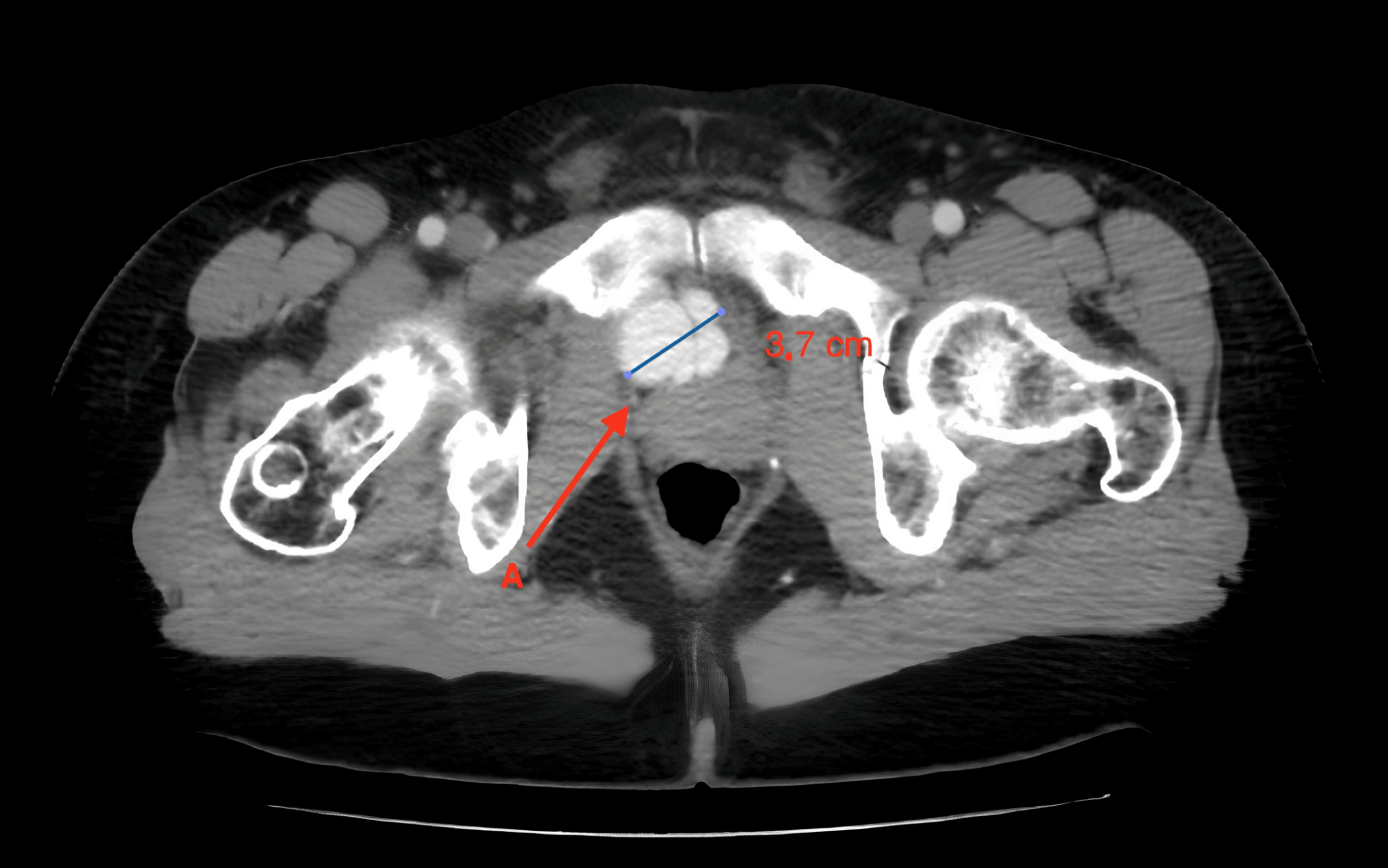
CT angiography. A horizontal section at the level of the symphysis. Up to 3.7 cm aneurysmatic confluent branches of the internal iliac vein can be noted (A).

**Figure 2:**
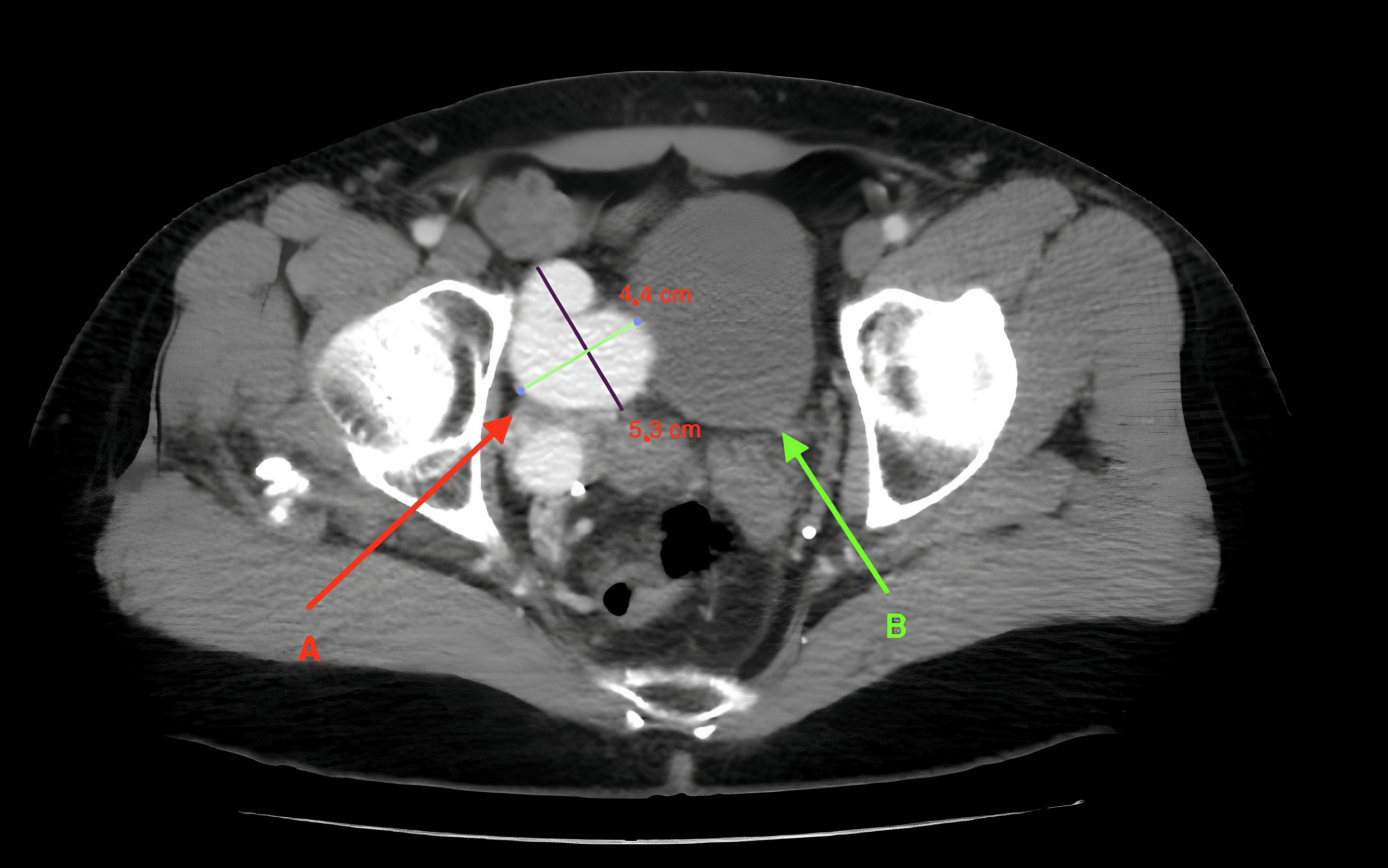
CT angiography. A horizontal section at the level of the lesser pelvis. Note the irregular aneurysmatic confluent branch of the right internal iliac vein (A) with a maximum diameter of 5.3 cm and the displacement of the right surface of the bladder (B) toward the midline.

**Figure 3:**
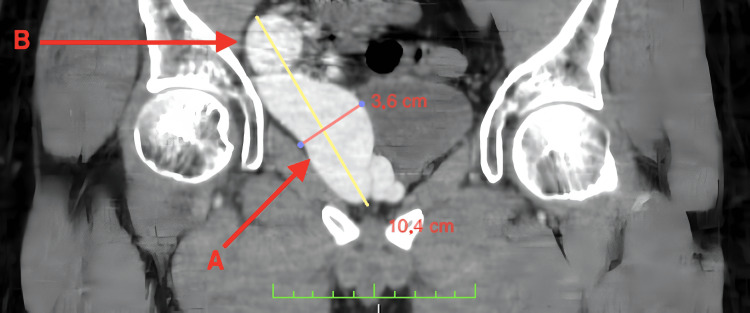
CT angiography. A coronal section of the pelvis. Note the craniocaudal extension of the venous aneurysm (A: confluent venous branch, B: internal iliac vein).

The CT angiography revealed distended serpentine venous trunks in the right lateral wall of the pelvic girdle. The largest had an indicative maximum diameter of 5.4 cm and a craniocaudal extension of slightly more than 10 cm. They showed more hyperdense imaging compared to the rest of the venous stems in the area (similar densitometric characteristics as arteries) and were in close anatomical contact with the right internal iliac artery. The entire picture was more compatible with arteriovenous communication which could not be recognized on the CT angiography. The distended venous trunks caused a pressure impression on the right lateral lower surface of the urinary bladder and were brought up to the area of the triangle of the urethra, causing mild pressure phenomena on the anterior fibromuscular layer of the prostate gland. In their descent at the level of the symphysis pubis, they extended marginally to the left of the midline (Figures 1-3).

Subsequently and after vascular surgery consultation, the patient underwent endovascular embolization (coiling) of the right internal iliac vein. The exact location of the arteriovenous communication could not be identified during the intervention, and the internal iliac vein was extendedly coiled. Two years after the endovascular procedure, the patient had not experienced significant relief from his symptoms. He still complained of the same dull pain in the right lesser pelvis and nocturia, which he had come to terms with over the years so that his quality of life felt no longer impaired.

**Figure 4:**
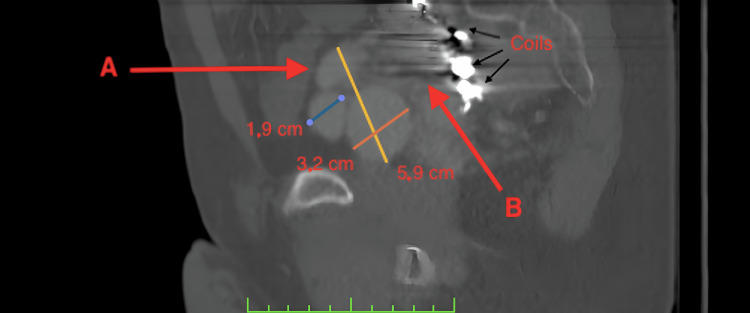
CT angiography. A sagital section of the pelvis. Note the endovascularly placed coils and the enormous right internal iliac vein (B), as well as the aneurysmatic venous confluent branch (A) two years after endovascular therapy.

**Figure 5:**
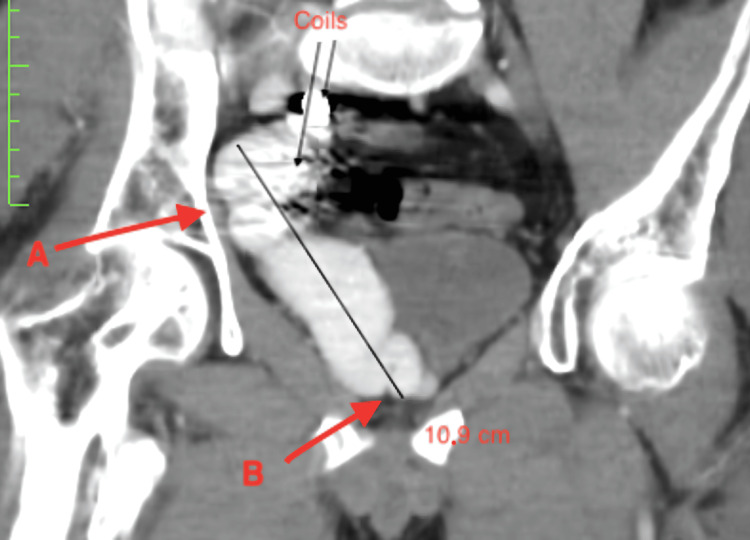
CT angiography. A coronal section of the pelvis (slight rotation of 10° to the right of the vertical axis). Note the coils, the enormous right internal iliac vein (A), and some of its tributaries (B) two years after endovascular therapy. Also, note the displacement of the bladder toward the midline.

The last CT angiographic follow-up two years after the embolization showed almost the same enormous venous aneurysms of the internal iliac vein and its confluent branches, as described above (Figures 4, 5).

As the endovascular procedure failed and the patient felt reasonably good about his quality of life, it has been decided to remain conservative at this time and monitor the patient regularly for follow-up checks.

## Discussion

In general, the presence of iliac vein aneurysms is characterized as a very rare clinical case due to their infrequent location [7]. These types of abnormalities are most commonly found in the external iliac vein, irrespective of the patient’s gender [8]. The development of an iliac vein aneurysm is mainly associated with an arteriovenous fistula due to trauma-related incidents. Even if arteriovenous fistulas are not approximate to the iliac area, they can still impact aneurysmal degeneration, as the lack of anterior muscular compression makes it an easy target [8]. Other than the arteriovenous fistulas, a smaller percentage of vein aneurysms are a result of proximal flow obstruction, while the rest are characterized as pure primary aneurysms [9]. Moreover, it is believed that iliac vein aneurysms, located mainly on the left side, could be connected with the presence of iliac vein compression syndrome. Some researchers support the idea that patients, especially endurance athletes, with vein compression syndrome are reported to have increased venous return, which can lead to aneurysm development [10].

Iliac vein aneurysms are also linked with the May-Thurner syndrome, which consists of an anatomical condition that results in compression of the left common iliac vein, between the right common iliac artery and the spine. It is possible that left deep vein thrombosis may be the result of this aforementioned compression [11]. Although deep vein thrombosis, as well as venous insufficiency, are the main signs of May-Thurner syndrome, in cases of venous outflow obstruction, aneurysmal degeneration in the external iliac vein can develop [8,12]. Therefore, in cases of aneurysmal degeneration of the external iliac vein, the presence of May-Thurner syndrome should be investigated. On the other hand, iliac vein aneurysms could be identified as primary, an entity that is much more common in women than men [8,13]. After the exclusion of other secondary causes of etiology, such as inferior vena cava anomalies and previous traumas, the aneurysm could be considered as primary [8].

It is important to understand that iliac vein aneurysms can present with multiple characteristics, which makes the diagnosis and identification challenging. Precisely, lower extremity pain, swelling, and insufficient venous flow are the key signs of this disease. Despite these prominent features, there are cases where patients do not experience any of the above-mentioned symptoms [14]. Various literature reviews warn that iliac vein aneurysm symptoms could be mistaken for an episode of deep vein thrombosis or pulmonary embolism. In other situations, professionals mistake them for soft tissue masses of the lower limbs. Furthermore, inguinal or femoral hernias are also a common misdiagnosis by the medical community [15].

In most of these cases, the aneurysm is identified incidentally during an evaluation for back or abdominal pain. Additionally, a strong correlation between venous aneurysms and pulmonary embolization has been reported by many studies. Specifically, in cases where venous blood stasis is observed, the formation of a thrombus typically follows [16]. Thus, in situations like these, pulmonary embolization may occur, creating a new potential connection between thrombosed limbs with venous insufficiency and pulmonary embolization. Finally, it should be noted that when dealing with such patients, medical professionals should consider iliac vein aneurysm as an extremely rare differential diagnosis [8]. In more severe situations, such venous aneurysms could lead to a profound shock due to a rupture in the intraperitoneal or retroperitoneal cavity. It should be remembered that retroperitoneal and intraperitoneal bleeding is a different medical entity from an iliac vein rupture [17,18].

Regarding treatment and management, assessment is performed through the use of advanced technological means, such as CT venography, duplex ultrasound, conventional venography, and magnetic resonance venography [19]. Despite its helpful diagnostic use, difficulties in ultrasound accuracy are often reported due to the location of the iliac aneurysm inside abdominal and pelvic cavities. Regardless of the plethora of options, one single method of diagnosis is yet to be decided, with venography being considered a strong candidate, owing to its ability to reveal and identify the exact location of the aneurysms. Arteriography is mostly used in cases of arteriovenous fistula-related iliac vein aneurysms to uncover crucial details [8].

Treatment options need to be contemplated according to the gravity of the complication, as well as other details of each case. Furthermore, surgical treatment is considered in most cases as the development of severe complications, such as rupture or hemorrhagic shock, is a potential danger [8]. Other reasons, in favor of this approach, are the potential morbidity, swelling, pain, as well as the presence of undefined mass [20].

The surgical approach consists of either open surgical methods or other endovascular management techniques. The decision regarding open surgery treatment is heavily influenced by the reason behind the aneurysm. Precisely, in aneurysms that are associated with arteriovenous fistulas, simple closure of the fistula could cease blood flow and regulate the aneurysm [21]. On the other hand, primary aneurysms require open surgical management to achieve aneurysm resection. Following this procedure, venous bypass or venography patch venoplasty is performed to reestablish the venous drainage [22,23]. As far as endovascular treatment methods are concerned, the available literature is relatively limited. Specifically, iliac aneurysms, as a result of arteriovenous fistula trauma, are most commonly managed by the use of arterial stent graft. In cases where open closure of arteriovenous fistulas is proposed, venous grafts convey a crucial role in the aneurysm exclusion [24]. Finally, internal iliac vein aneurysms, of primary origins, are managed by coil embolization [15]. In situations where a conservative approach is preferred, the patients undergo interval follow-up, as well as anticoagulation regulation.

## Conclusions

Undoubtedly, the internal iliac vein is a complex and intricate-to-study short blood vessel that serves many significant functions in the human body. Iliac vein aneurysms are considered a rare clinical entity requiring delicate and careful diagnosis, as well as thoughtful treatment. Identification of such cases has been made easier owing to advanced technological imaging techniques, such as CT and venography. Finally, treatment options need to be decided after cautious planning, as venous aneurysms require a different approach according to their characteristics and features.
